# ANTXR1 Is a Prognostic Biomarker and Correlates With Stromal and Immune Cell Infiltration in Gastric Cancer

**DOI:** 10.3389/fmolb.2020.598221

**Published:** 2020-12-15

**Authors:** Xiaodong Huang, Jie Zhang, Yongbin Zheng

**Affiliations:** ^1^Department of General Surgery, The Third People's Hospital of Hubei Province, Wuhan, China; ^2^Department of Obstetrics and Gynecology, Renmin Hospital of Wuhan University, Wuhan, China; ^3^Department of Gastrointestinal Surgery, Renmin Hospital of Wuhan University, Wuhan, China

**Keywords:** gastric cancer, immune infiltration, immunotherapy, bioinfomatics, ANTXR1

## Abstract

Gastric cancer (GC) is a complex and heterogeneous disease, making it difficult to ascertain the optimal therapeutic approach for individual GC patients. Stromal and immune cell infiltration in GC has a strong correlation with clinical outcomes; however, the underlying mechanisms that drive immunosuppression remain vastly undiscovered. Recent studies validated that anthrax toxin receptor 1 (ANTXR1) is aberrantly expressed in several cancers and holds promise as a new therapeutic target for cancer. However, its immunological roles in GC are still unclear. Here, we show that we identify the distinct stromal and immune cell infiltration in GC between the high and low ANTXR1 expression group by analyzing genomic data. Clinically, ANTXR1 is highly expressed in GC and correlates with adverse clinicopathological characteristics. Additionally, high ANTXR1 expression is linked to markedly poor clinical outcomes and resistance to chemotherapy, whereas the low ANTXR1 expression group is correlated with better outcomes and response to chemotherapy in GC patients. We further revealed the differential landscape of somatic tumor mutation burden (TMB) between the two groups and observed that patients with high ANTXR1 expression suffered from a lower TMB, potentially leading to less sensitivity to checkpoint therapy. Molecularly, results displayed that ANTXR1 is an immunosuppressive element, which may perform its function via promoting the secretion of immunosuppressive factors that play a significant role in modulating tumor-associated fibroblast transformation, M2 macrophage polarization, and T cell exhaustion. Gene set enrichment analysis revealed that cancer-related pathways including epithelial-to-mesenchymal transition, focal adhesion, and transforming growth factor-β (TGF-β) signaling pathways were enriched in high ANTXR1 expression tumors. Our work suggests that ANTXR1 could not only serve as a valuable prognostic biomarker in GC but also be deemed as a potential immunotherapeutic target and useful biomarker of sensitivity to chemotherapy.

## Introduction

Gastric cancer (GC), imposing a considerable global health burden, is the fifth most common tumor and the third leading cause of cancer-associated death in the world (Bray et al., 2018). Despite advances in screening strategies for early detection, they are neither fulfilled nor feasible in many areas of the world, leading to delayed diagnosis in most patients (Ajani et al., 2017). Even with improvements in endoscopic, surgical, and comprehensive treatments, however, the global 5-years survival rates range from 25 to 30% (Ajani et al., 2017) remain virtually unsatisfactory, with the exception of those in Japan and South Korea (>50%) (Torre et al., 2016). Adequate surgical resection followed by adjuvant chemoradiotherapy is the common treatment for resectable GC at advanced stages (Van Cutsem et al., 2016). Targeted therapies including monoclonal antibodies against human epidermal growth factor receptor 2 (HER2) (Roviello and Generali, 2018), anti-epidermal growth factor receptor (anti-EGFR) (Maron et al., 2018), and immune checkpoint blocker such as programmed cell death protein-1 (PD-1) inhibition (Kim et al., 2018) may prolong survival in selected patients. The majority of GC patients at advanced stages, however, will suffer local recurrences and remote metastases after initial surgery (Niccolai et al., 2015). Meanwhile, an unavoidable issue is that resistance to chemotherapy and/or radiotherapy, as a consequence of considerable clinicopathologic and genomic heterogeneity, distinct molecular characteristics, and the complex immunosuppressive microenvironment that surrounds the tumor niche, has moderately emerged in GC patients (Bijlsma et al., 2017; Roma-Rodrigues et al., 2019). Growing evidences suggest that the immune system performs its crucial function during tumorigenesis and progression (Gentles et al., 2015), and much attention has been paid to immunotherapy because of the attracting clinical outcomes. Despite the unprecedented success of immunotherapy, the majority of patients with carcinoma have little benefit from it, highlighting the urgent need to identify new molecules that could be developed in the next generation of immunotherapy. Therefore, it is important to determine novel immunotherapeutic targets and prognostic markers of GC to shed light on the stratification of patients who may respond to immunotherapy.

Anthrax toxin receptor 1 (ANTXR1), otherwise known as tumor endothelial marker 8 (TEM8), is a highly conserved transmembrane glycoprotein overexpressed on tumor vasculature (Chaudhary et al., 2012). ANTXR1 can facilitate the entrance of anthrax toxin into cells and has been demonstrated to be overexpressed in several cancer types including gastric, breast, colon, and pancreatic tumors (Rmali et al., 2004; Davies et al., 2006; Szot et al., 2018; Alcala et al., 2019). Previous research also revealed that anti-TEM8 antibody–drug conjugate is promising in augmenting therapies in diverse cancer types based on preclinical study (Szot et al., 2018). Moreover, Byrd TT and colleagues developed anti-ANTXR1 chimeric antigen receptor T cell for targeted therapy of triple-negative breast cancer and successfully induced regression of established patient-derived xenograft tumors (Byrd et al., 2018). Recently, Sotoudeh et al. reported that ANTXR1 was overexpressed in GC and a promising molecular target for both clinical and preclinical assessment for immunotherapy of stomach cancer (Sotoudeh et al., 2019). The above findings implicate that ANTXR1 plays a vital role in tumor angiogenesis, progression, invasion and metastasis, thus making it a potential immunotherapeutic target. However, the potential mechanisms of ANTXR1 in gastric tumor progression and cancer immunology have not been extensively studied.

In the current study, we comprehensively investigated associations between ANTXR1 expression and clinical outcomes of GC patients based on two independent datasets. It is widely accepted that the stroma of tumor microenvironment (TME) is correlated with the resistance of chemotherapy of cancer; thereby, we evaluated whether ANTXR1 is a predictor for GC patients' response to chemotherapy. We further assessed the differential landscape of somatic tumor mutation burden (TMB) between the two groups (high vs. low ANTXR1 expression). Importantly, we analyzed the correlation of ANTXR1 with infiltration level of stromal and immune cells in the TME. The findings in the present research not only shed light on the crucial role that ANTXR1 plays in TME of GC but also afford potential molecular functions and mechanisms of ANTXR1 in stromal or immune cell infiltration.

## Materials and Methods

### Acquisition of Datasets

Two cohorts of GC patients from two independent databases were included in this study. Level 3 gene expression RNA-sequencing data [log2(normal_count+1)] of The Cancer Genome Atlas (TCGA) stomach adenocarcinoma (STAD) patients, including 415 tumor tissues and 35 adjacent normal controls, were collected from the UCSC Xena browser (https://xena.ucsc.edu/). Clinical information, such as age, gender, histological grade, disease stage, Lauren classification, and clinical outcomes of TCGA-STAD samples, was obtained from the Genomic Data Commons (https://gdc.cancer.gov/). For Gene Expression Omnibus (GEO) data, gene expression data of 300 GC patients in Asian Cancer Research Group (ACRG) cohort (GSE62254) were downloaded from the GEO database using *GEOquery* (V2.54.1) R package. The annotation platform GPL570 (Affymetrix Human Genome U133 Plus 2.0 Array) was utilized to annotate gene names. Clinical data of 300 patients with GC in the ACRG cohort were acquired from a previous study (Cristescu et al., 2015).

### Differentially Expressed Genes Analysis

According to the median of ANTXR1 expression, GC patients were divided into two groups (high and low). The R package *limma* (V3.42.0), which performs an empirical Bayesian approach to evaluate gene expression changes using the moderated *t*-test (Ritchie et al., 2015), was applied to identify differentially expressed genes (DEGs) in ANTXR1 expression subgroups. The adjusted *P* < 0.05 and |log_2_(fold change)| > 1 were deemed to be the cutoff criteria to screen for DEGs.

### Gene Ontology and Pathway Enrichment Analysis

By applying *clusterprofiler* (V3.14.0) R package (Yu et al., 2012), we conducted gene ontology (GO) functional analysis. Enriched GO terms with *P* < 0.05 and false discovery rate (FDR) < 0.05 were considered as statistical significance. To further explore the underlying pathway of the DEGs in GC, we performed gene set enrichment analysis (GSEA) of the upregulated genes. Gene sets “c2.cp.kegg.v7.1.entrez.gmt” and “h.all.v7.1.entrez.gmt,” which summarize and represent well-defined biological states and pathway processes, were obtained from the Molecular Signatures Database (MSigDB) of Broad Institute (https://www.gsea-msigdb.org/gsea/msigdb). Enriched *P* values were based on 10,000 permutations and then multiple tests were adjusted using the Benjamini–Hochberg program to control the FDR. Enriched signaling pathways with *P* < 0.05 and FDR < 0.05 were defined as statistical significance.

### Analysis of Stromal and Immune Infiltration

Stromal, immune, and ESTIMATE scores were obtained by applying the ESTIMATE (Estimation of STromal and Immune cells in MAlignant Tumors using Expression data) algorithm (Yoshihara et al., 2013). In addition, TIMER (Tumor Immune Estimation Resource) (http://cistrome.org/TIMER/) was used to carry out comprehensive correlation analysis between tumor-infiltrating immune cell signatures and ANTXR1 expression. In addition, another approach to evaluate infiltration levels of stromal and immune cells adopted in the present study was xCell, which infers the fraction of 64 immune and stromal cell types in tumor samples based on gene signatures (Aran et al., 2017). Correlation between ANTXR1 expression and the infiltration levels of 64 immune and stromal cell types in each tumor sample was computed.

### Estimates of Somatic Mutations

We obtained all mutation data from 395 patients in the TCGA-STAD cohort consisting of deletions, insertions, and substitutions across bases and divided the data into two groups according to the ANTXR1 expression. Mutation profiling of the two groups by waterfall plot was conducted by using R Bioconductor package *Maftools* (V2.2.10) (Mayakonda et al., 2018). Moreover, the following formula: TMB = (total count of variants)/(the whole length of exons) was employed to calculate TMB for each GC patients.

### Statistical Analysis

Unpaired two-samples *t*-test and paired-samples *t*-test were, respectively, adopted to compare the expression level of ANTXR1 in unpaired samples and paired samples. The unpaired Wilcoxon test was employed to compare ranked data with two categories, and the Kruskal–Wallis test was used for comparisons among three or more groups. Kaplan–Meier survival analysis was performed to evaluate the prognostic value of ANTXR1 based on the TCGA-STAD cohort or ACRG cohort using *survival* (V3.1-8) R package. In addition, Cox proportional hazards models were generated to reveal the independent indicators associated with overall survival (OS) or disease-free survival (DFS) using *survminer* (V0.4.6) R package. Log-rank test was used to test the association. Patients from the ACRG cohort were separated into two groups according to the optimal cutoff value of ANTXR1 expression level based on survival analysis. By applying data from patients in the ACRG cohort, a Cox proportional hazards model was generated to evaluate the intensity of the interaction between the two groups and adjuvant chemotherapy. The interaction test was implemented by likelihood ratio test. All statistical analyses were conducted in R, Version 3.6.1 (http://www.r-project.org), and *P* < 0.05 was declared as statistically significant.

## Results

### Overexpression of ANTXR1 Correlated With Tumor Aggravation in GC

**Figure 1:**
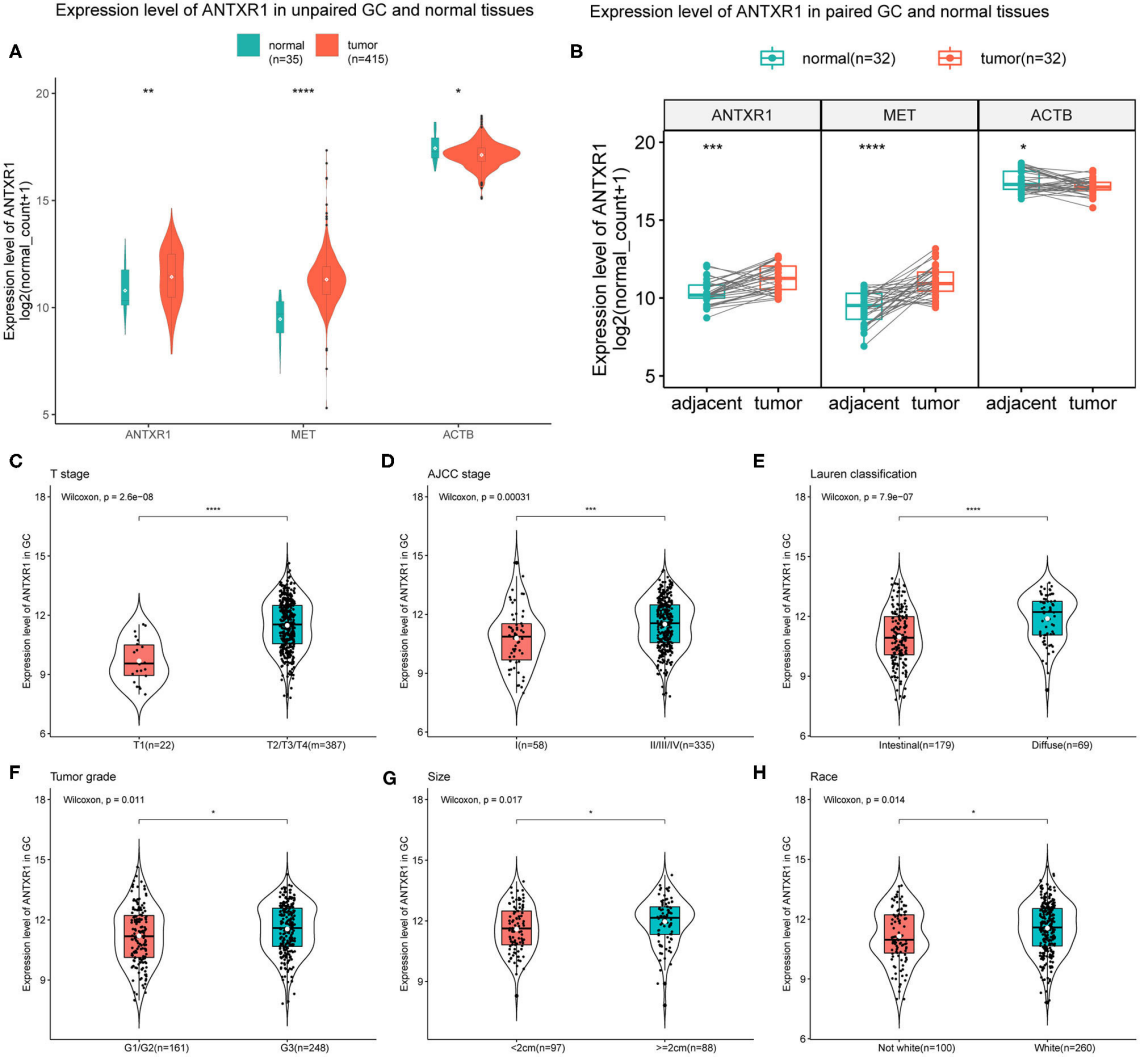
Elevated expression level of ANTXR1 correlated with aggravation of gastric cancer. **(A)** Expression level of ANTXR1 in unpaired tumor and adjacent tissues. **(B)** Expression level of ANTXR1 in unpaired tumor and adjacent tissues. MET and ACTB gene expression were considered as positive and negative control, respectively. **(C–H)** Comparisons of ANTXR1 expression levels in different clinical features in GC. **(C)** T stage, **(D)** AJCC stage, **(E)** Lauren classification, **(F)** histological grade, **(G)** tumor size, and **(H)** race. Box plots elements (center line, median; box boundaries, upper and lower quartiles; whiskers, maximum and minimum). *P < 0.05, **P < 0.01, ***P < 0.001, ****P < 0.0001.

To assess the expression level of ANTXR1 in patients with GC, we compared the expression level of ANTXR1 in non-paired tumor and adjacent tissues and paired samples, and the MET was considered as positive control while ACTB was considered as negative control. Results showed that there was a significant difference in the expression of ANTXR1 between non-paired/paired gastric tumors and adjacent samples (Figures 1A,B). Using Wilcoxon test, we further investigated the expression level of ANTXR1 in different clinicopathological features of GC patients and found that its level significantly correlated with T stage (*P* < 0.0001), AJCC stage (*P* < 0.001), Lauren classification (*P* < 0.0001), WHO grade (*P* < 0.05), tumor size (*P* < 0.05), and race (*P* < 0.05) based on the TCGA dataset (Figures 1C–H). The above results implicated that ANTXR1 is significantly overexpressed in stomach cancer and may be associated with tumor progression.

### ANTXR1 Expression Is Involved in Outcomes of GC

**Figure 2:**
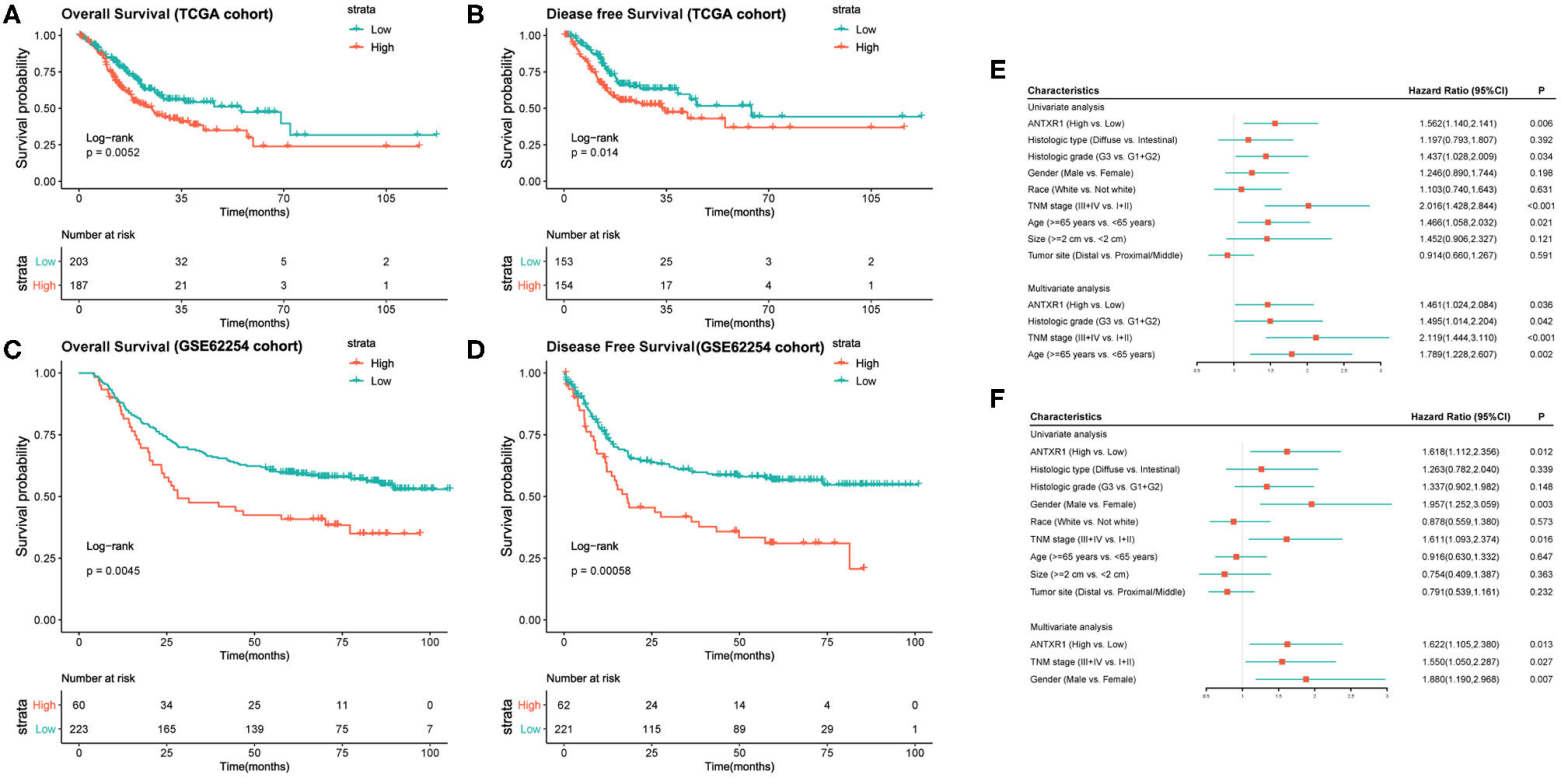
High expression of ANTXR1 predicts adverse clinical outcomes of gastric cancer patients by applying Kaplan–Meier survival analysis. **(A,B)** Based on TCGA datasets. **(C,D)** Based on GSE62254 cohort. Univariate and multivariate analysis of clinical parameters associated with overall survival **(E)** and disease-free survival **(F)**.

Since we found that ANTXR1 was aberrantly expressed in GC and correlated with tumor progression, its prognostic value was further investigated. We divided the patients into two groups according to the optimal cutoff, and the prognostic significance of this gene was estimated by applying Kaplan–Meier survival analysis utilizing data from both TCGA and ACRG cohorts. The results suggested that high ANTXR1 expression is significantly correlated with not only poor OS but also DFS in GC patients based on TCGA cohort (Figures 2A,B), which was consistent with results obtained from the ACRG cohort (Figures 2C,D). In addition, by utilizing the TCGA dataset, univariate and multivariate Cox regression analyses were conducted to assess the independent prognostic value of ANTXR1 in GC. Results showed that ANTXR1 is an independent indicator associated with poor OS [hazard ratio (HR):1.461; 95% confidence interval (CI):1.024, 2.084; *P* = 0.036] as well as DFS (HR = 1.622; 95% CI: 1.105, 2.380; *P* = 0.013) in GC patients from the TCGA database (Figures 2E,F). Taken together, these findings demonstrated that ANTXR1 is an independent prognostic indicator, and overexpression of ANTXR1 predicts unfavorable prognosis for GC patients.

### Identification of DEGs and Functional Annotation

**Figure 3:**
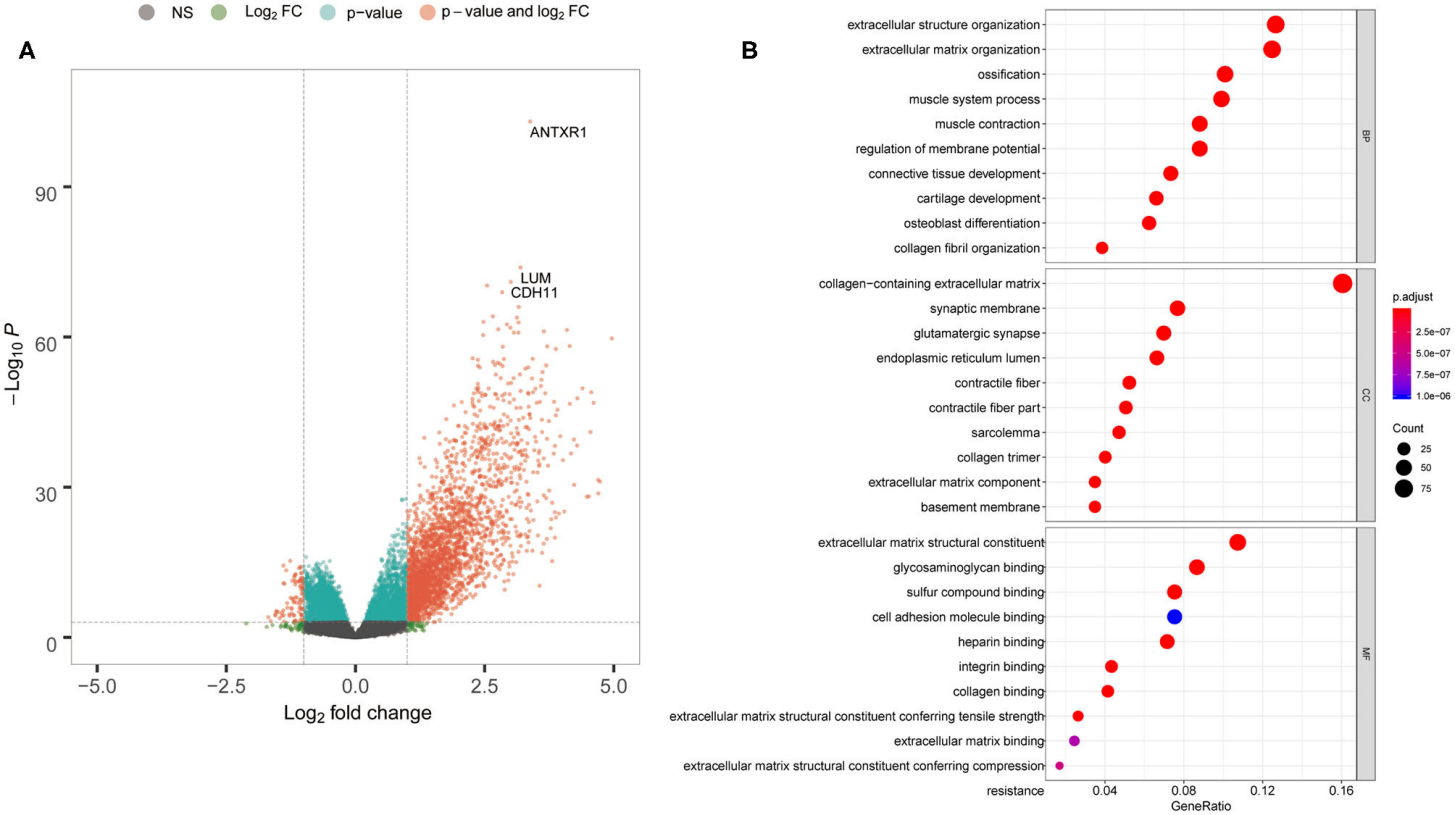
ANTXR1 involved in definite gene ontology (GO) terms in gastric cancer. **(A)** Volcano plot of the differentially expressed genes. **(B)** Results of BF (biological function), CC (cellular component), and MF (molecular function) terms analyzed.

To explore DEG profiles from the TCGA cohort with high and/or low ANTXR1 expression, we performed expression profiles analysis of 415 GC cases. In total, 2,630 genes were upregulated and 130 genes were downregulated in high ANTXR1 expression than the low ANTXR1 expression group using the R package *limma*. The representatives of the upregulated genes are shown as heatmap (Supplementary Figure 1 and Supplementary Table 1) and volcano plot (Figure 3A). The identified 2,635 upregulated genes were subjected to GO enrichment analysis utilizing *clusterProfiler* R package. The top 10 terms of enriched biological processes (BP), molecular function (MF), and cell component (CC) are shown in Figure 3B. We found that the upregulated genes were mostly related to extracellular matrix, which implicated that these genes might function as key regulators in the stroma of GC.

To explore the potential pathways of the upregulated genes in GC, we further conducted GSEA using the MSigDB hallmark gene sets and KEGG pathway gene sets. Interestingly, among the most significantly enriched KEGG pathway gene sets, two groups of cancer-associated gene sets including focal adhesion and TGF-beta signaling pathway were highly enriched in patients harboring high ANTXR1 expression level (Supplementary Figure 2A). For the hallmark gene sets, epithelial mesenchymal transition (EMT) gene set was significantly enriched in the high ANTXR1 expression group (Supplementary Figure 2B). Collectively, these findings indicated that ANTXR1 may participate in regulating the tumor stromal environment and promotes tumor metastasis.

### Correlation Between ANTXR1 and Immune and Stromal Cell Infiltration in GC

**Figure 4:**
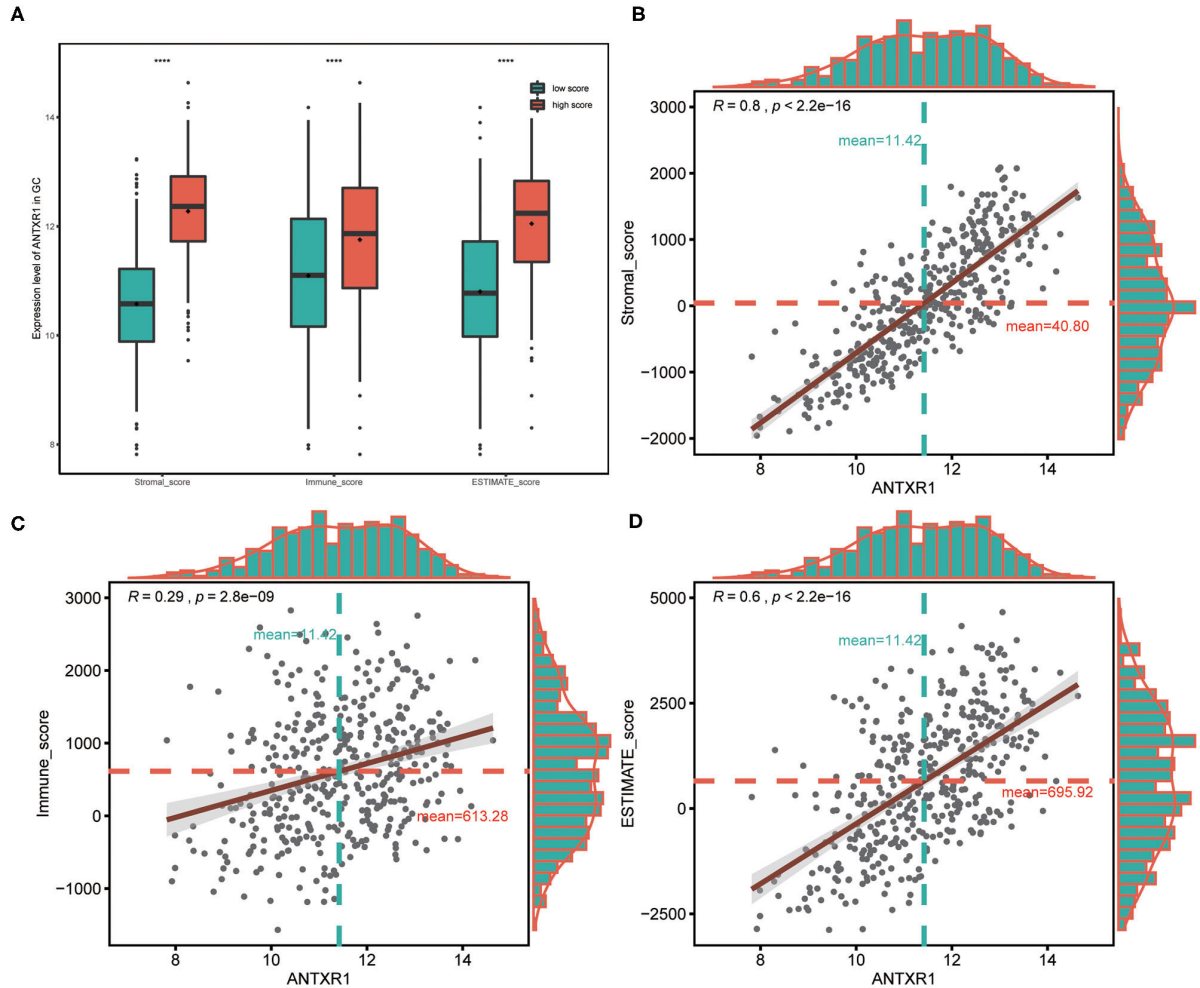
Association between ANTXR1 expression and ESTIMATE score in gastric cancer. **(A)** High ANTXR1 expression associated with high immune score, stromal score, and ESTIMATE score in gastric cancer patients. Box plot elements (center line, median; box boundaries, upper and lower quartiles; whiskers, maximum and minimum). ANTXR1 expression was positively correlated with stromal score **(B)**, immune score **(C)**, and ESTIMATE score **(D)** in gastric cancer patients. ****P < 0.0001.

**Figure 5:**
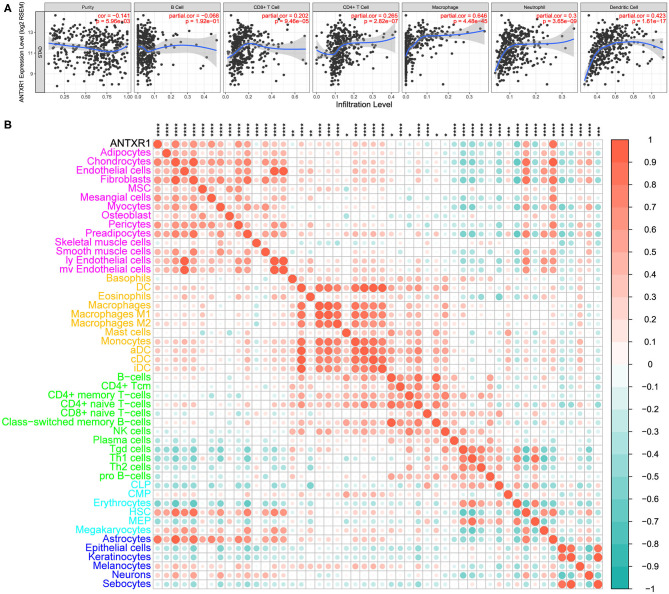
Correlation between ANTXR1 expression and immune and stromal cell infiltration in gastric cancer. **(A)** Relationship between ANTXR1 expression and six immune cells conducted by TIMER. **(B)**. Corrplot of ANTXR1 expression and 49 cell types calculated by xCell in gastric cancer. *P < 0.05, **P < 0.01, ***P < 0.001, ****P < 0.0001.

In order to assess the immunological role of ANTXR1 play in GC, we further investigated its association with immune and stromal cell infiltration in GC. Firstly, we compared the differences of immune score, stromal score, and ESTIMATE score between high ANTXR1 expression group and low. The findings showed that stromal score, immune score, and ESTIMATE score are significantly higher in patients with high ANTXR1 expression than those with low ANTXR1 expression (Figure 4A). Additional linear regression analyses also indicated that ANTXR1 expression has positive correlations with immune score, stromal score, and ESTIMATE score (Figures 4B–D), which implied that it has a notable impact on infiltration levels of stromal and immune cells. To better understand the relationship between ANTXR1 expression and the TME, TIMER algorithm was employed to analyze the correlation of ANTXR1 expression with tumor purity and six kinds of tumor-infiltrating immune subsets in STAD samples. The results of the correlation analyses showed that CD8+ T cells, CD4+ T cells, macrophages, neutrophils, and dendritic cells were positively correlated with the expression of ANTXR1, while they were negatively correlated with tumor purity (Figure 5A). Moreover, another algorithm (xCell) was also performed to investigate the predominant cell types that function in this process. Correlations between ANTXR1 expression and 64 non-cancerous cell types were investigated based on GC patients in TCGA cohort. The results indicated that a total of 49 cell types significantly correlated with ANTXR1 expression (Figure 5B), among them 30 types were positively correlated while 19 types were negatively correlated. The 49 cells types consist of 12 lymphoid cells, 11 myeloid cells, 14 stromal cells, six stem cells, and six other cell types. Intriguingly, we acquired that the expression profiles of the majority of stromal cells and myeloid cells have robust correlations with ANTXR1 expression in STAD patients, whereas the bulk of lymphoid cells have negative correlations. These results powerfully suggested that ANTXR1 plays a crucial role in infiltration levels of immune and stromal cells in GC.

### Correlation Analysis Between ANTXR1 and Immunosuppressive Properties

**Figure 6:**
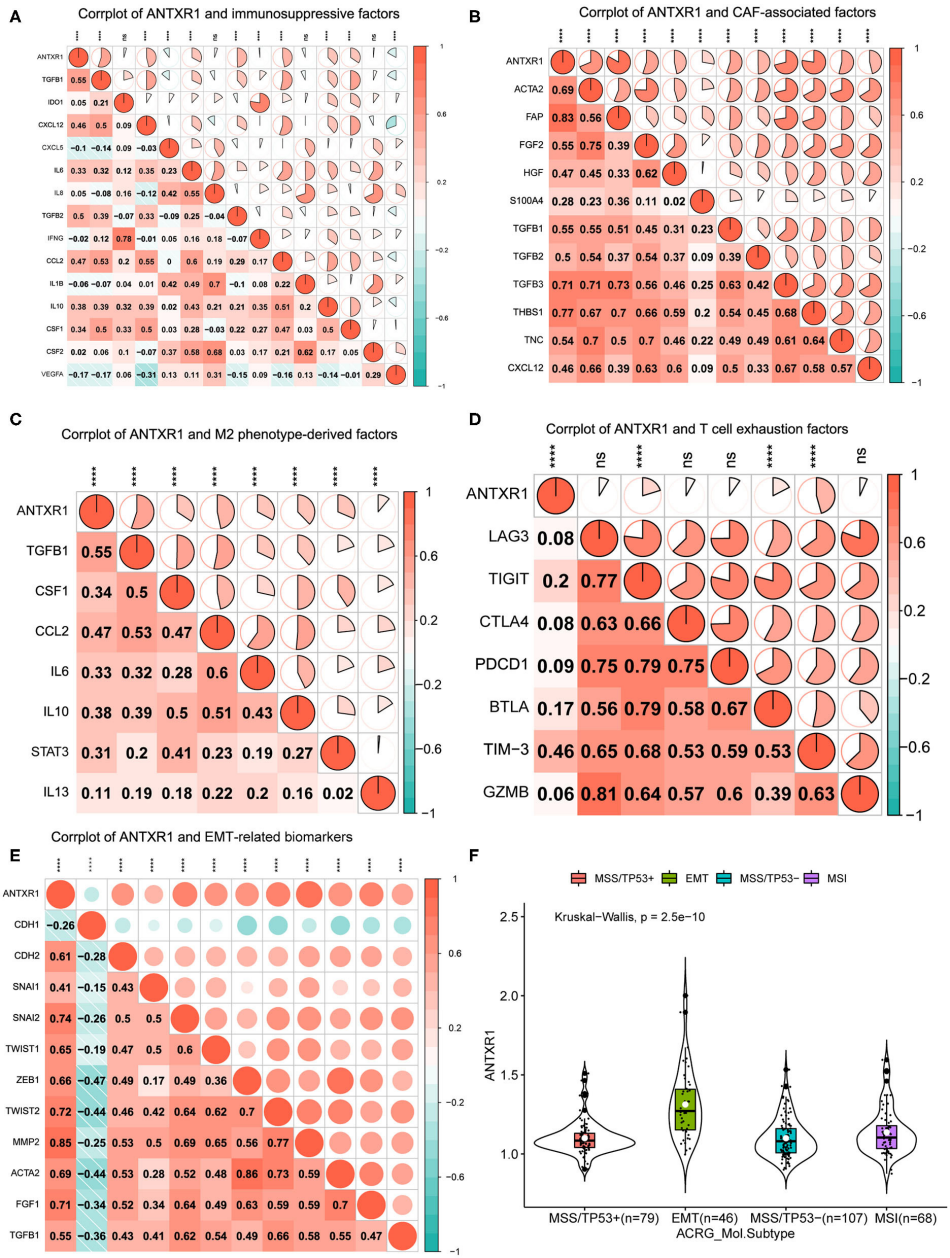
ANTXR1 contributes to immunosuppression and prompt EMT. **(A)** Corrplot between ANTXR1 and immunosuppressive cell recruitment factors. **(B)** Corrplot between ANTXR1 and cancer-associated fibroblast (CAF)-related factors. **(C)** Corrplot between ANTXR1 and M2 phenotype-driving factors. **(D)** Corrplot between ANTXR1 and exhausted T cell receptors. **(E)** Corrplot between ANTXR1 and common biomarkers of EMT. **(F)** Box plot of ANTXR1 expression levels in different ACRG molecular subtypes. Box plot elements (center line, median; box boundaries, upper and lower quartiles; whiskers, maximum and minimum). EMT, epithelial–mesenchymal transition; ACRG, Asian Cancer Research Group. *P < 0.05, **P < 0.01, ***P < 0.001, ****P < 0.0001, ns *P* > 0.05.

Since primary analyses indicated that ANTXR1 was predominantly positively correlated with immunosuppressive cells, such as fibroblasts and M2 macrophages, we hypothesized that ANTXR1 could participate in regulation of the immunosuppressive factors of GC. For the sake of validating this, we conducted a correlation analysis of ANTXR1 expression and pivotal factors that drive the recruitment of myeloid-derived suppressor cells, cancer-associated fibroblasts (CAFs), and tumor-associated macrophages (TAMs), as well as the immunosuppressive factors these cells secreted. As shown in Figure 6A, ANTXR1 was significantly positively associated with the majority of immunosuppressive factors. CAFs are involved in tumor initiation and progression by promoting angiogenesis, survival of malignant tumor cells, EMT, as well as cell proliferation via secretion of soluble factors (Huang et al., 2014). In this study, CAF-associated factors were found to be significantly correlated with ANTXR1 expression (Figure 6B). TAMs are composed of M1 and M2 TAMs, and it is M2 that plays a vital role in tumor progression, promoting immunosuppressive signal in the TME (Gambardella et al., 2020). Indeed, we found that critical factors driving M2 phenotype differentiation were strongly or moderately related to ANTXR1 expression (Figure 6C).

It is well-established that T cell exhaustion has crucial implications for the success of immune checkpoint blockade and adoptive T cell transfer treatments (Blank et al., 2019). To further analyze relationships between marker genes of T cell exhaustion and ANTXR1 expression, results of correlation analyses revealed that TIM-3, TIGIT, and BTLA have significant correlations with ANTXR1 expression (Figure 6D). Intriguingly, TIM-3, as an important gene that modulates T cell exhaustion, is moderately positively correlated with ANTXR1 expression, manifesting that ANTXR1 plays a pivotal role in TIM-3 mediating T cell exhaustion.

EMT was well-known for the critical role it plays in embryogenesis and some other pathophysiological processes, especially tumor metastasis (Huang et al., 2015). Since primary analyses indicated that the EMT gene set was significantly enriched in the high ANTXR1 expression group, we therefore analyzed whether ANTXR1 expression has correlations with common EMT biomarkers. The correlation analysis showed that EMT biomarkers, except CDH1, were dramatically positively correlated with ANTXR1 expression (Figure 6E). Specifically, ANTXR1 has a positive relation to mesenchymal cell and a negative correlation with epithelial cell markers. Besides, analysis of ANTXR1 expression level in patients with different ACRG molecular subtypes also revealed that ANTXR1 expression was notably higher in patients with EMT signature than those with other subtypes (Figure 6F). Taken together, these results further confirmed the findings that ANTXR1 plays a crucial role in regulation of TME in GC, which functions by driving the recruitment of immunosuppressive cells to secrete soluble factors, modulating M2 and CAF transformation, mediating T cell exhaustion, and prompting EMT; this can ultimately motivate tumor immune evasion, leading to adverse clinical outcomes for GC patients.

### High ANTXR1 Expression Contributes to Chemotherapy Resistance

**Figure 7:**
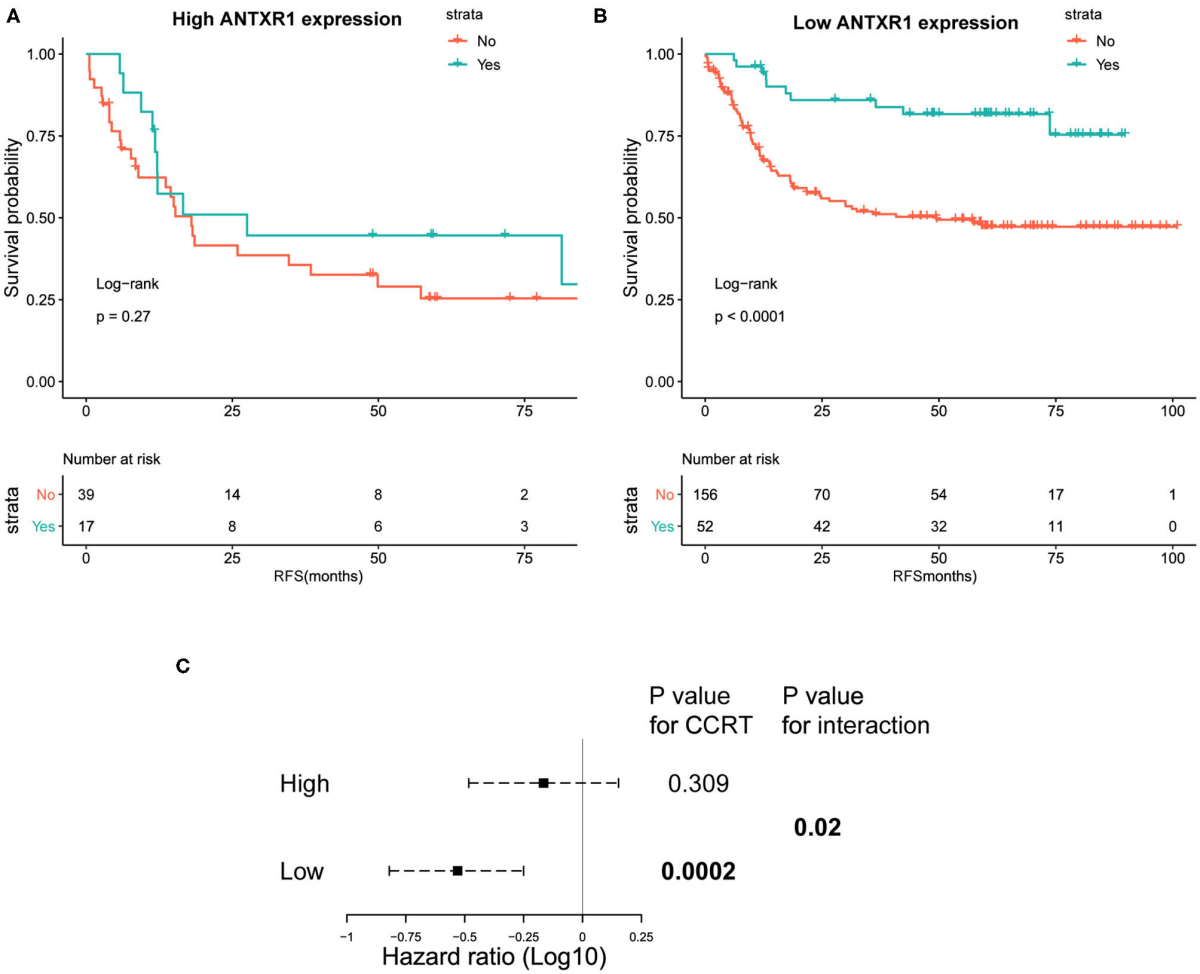
Kaplan–Meier plots of recurrence-free survival (RFS) analysis. **(A,B)** Kaplan–Meier plots of RFS among patients who received CTX and those who did not for high and low ANTXR1 expression group. Log-rank test was utilized to determine the *P*-values. **(C)** Interaction of ANTXR1 expression with CTX in patients with gastric cancer. The Cox proportional hazards regression model was employed to analyze the interaction between ANTXR1 expression and CTX. CTX, adjuvant chemotherapy.

Considering that the adjuvant chemotherapy information was available for patients from the ACRG cohort, we further sought to explore whether ANTXR1 expression level was correlated with different clinical outcomes for adjuvant chemotherapy. These patients were divided into a high and low ANTXR1 expression group, and the difference in recurrence-free survival (RFS) rates was independently compared. Adjuvant chemotherapy was found to prolong the RFS time in patients with low ANTXR1 expression (*P* < 0.0001 by log-rank test; Figure 7B). Compared to those who did not receive adjuvant chemotherapy, the HR for recurrence among those who received was 0.33 (95% CI: 0.18–0.62, *P* < 0.001 by likelihood ratio test). However, patients with high ANTXR1 expression did not benefit or only benefitted a little from adjuvant chemotherapy (*P* = 0.27 by likelihood ratio test; Figure 7A). Additionally, we conducted an interaction test to evaluate the real difference between the two groups in the light of the influence of adjuvant chemotherapy. We performed the Cox proportional hazard regression model, and the interaction of the high and low ANTXR1 expression groups with adjuvant chemotherapy achieved statistical significance (*P* = 0.02 by likelihood ratio test; Figure 7C), suggesting that patients with high ANTXR1 expression benefit less from adjuvant chemotherapy than patients with low ANTXR1 expression.

### Somatic Mutation Analysis of the Two Groups

**Figure 8:**
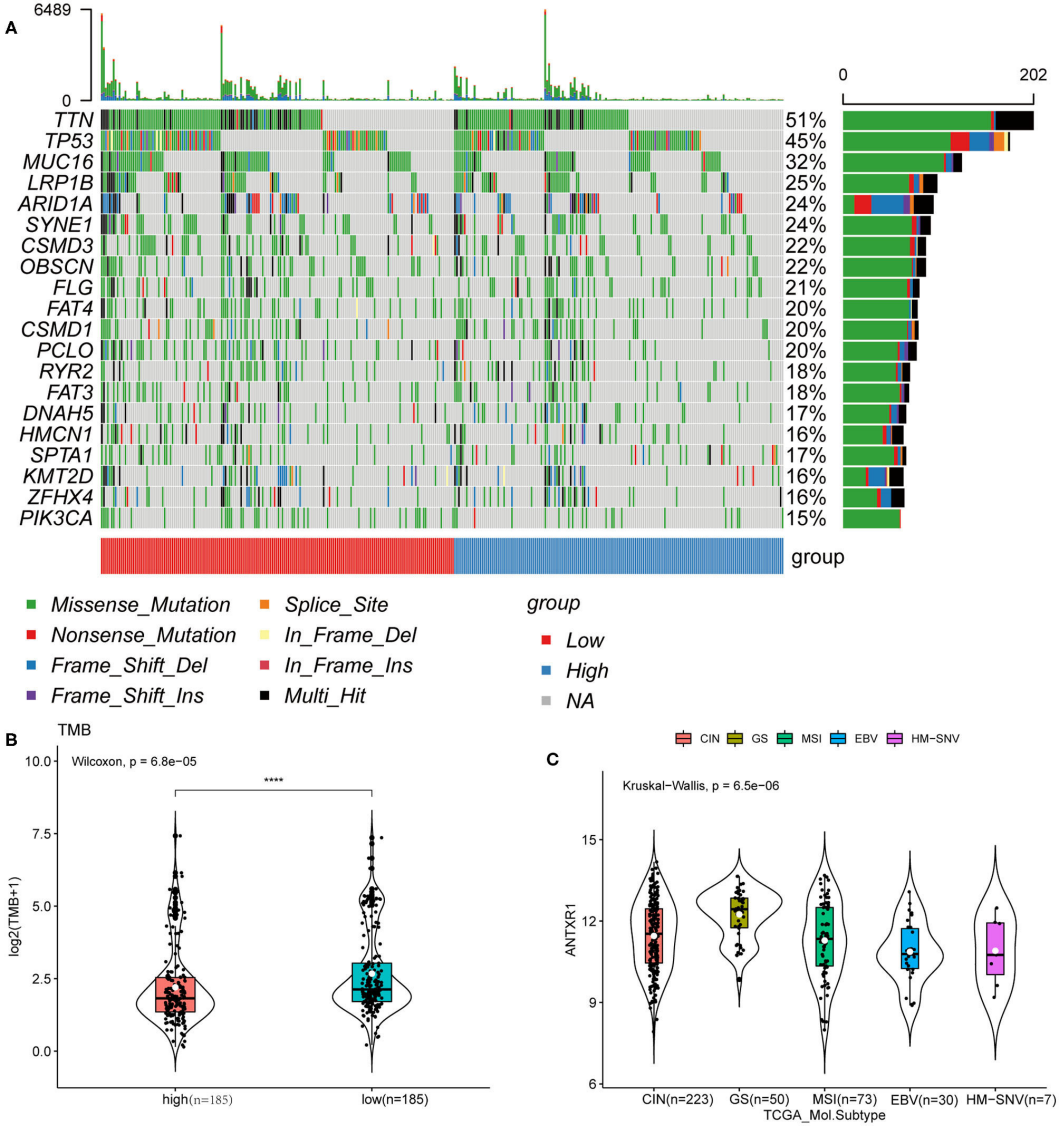
ANTXR1 expression is correlated with TMB in gastric cancer. **(A)** Distribution of the top 20 variant mutated genes between high ANTXR1 expression and low was shown in the waterfall plot. **(B)** Association between TMB and ANTXR1 expression. **(C)** Distribution of ANTXR1 expression levels of TCGA molecular subtypes. Box plot elements (center line, median; box boundaries, upper and lower quartiles; whiskers, maximum and minimum). TMB, tumor mutation burden. ****P < 0.0001.

We next investigated mutational variants of patients from the TCGA-STAD cohort. The somatic mutation landscape of the two groups is shown in Figure 8A. The evaluation of the TMB of the two groups revealed that TMB in patients with high ANTXR1 expression were significantly lower than those with low ANTXR1 expression (*P* < 0.001; Figure 8B). In addition, we assessed the association between ANTXR1 expression and known TCGA molecular subtypes (CIN, EBV, GS, HM-SNV, and MSI subtype) (Liu et al., 2018). Among the five subtypes, the average ANTXR1 expression of the GS subtype was the highest (*P* < 0.0001; Figure 8C). Previous studies reported that high TMB and MSI, leading to generation of neoantigens, are typical indicators predicting the sensitivity to immune checkpoint inhibitor treatment (Chan et al., 2019; Samstein et al., 2019). Taken together, these results suggest that patients with low ANTXR1 expression may be more sensitive to immune checkpoint inhibitor therapy.

## Discussion

GC is a heterogeneous disease with multitudinous clinicopathologies and genotypes (Ajani et al., 2017), thereby making it arduous to ascertain the optimum approach for an individual GC patient. Even with surgery in combination with adjuvant chemotherapy, however, numerous advanced GC patients will suffer a rapid relapse due to the extremely invasive feature of this illness. Cancer immunotherapy, which can modulate the immune system to struggle against cancer, has recently emerged as a revolutionary and promising therapeutic strategy of multiple tumor types including GC. Several immunotherapeutic drugs have been applied for the clinical treatment of GC; however, unpredictable patient response rates and underlying immune-related adverse effects remain two outstanding questions. One feasible therapeutic methodology is to exploit cancer immunotherapy for GC by targeting molecules that overexpressed in cancer cells, which plays a pivotal role in immunosuppressive microenvironment.

ANTXR1 has been reported to participate in tumor progression in numerous kinds of carcinomas, and its overexpression in tumor cells makes it a promising prognostic biomarker and therapeutic target accordingly (Chaudhary et al., 2012). In the current study, we found that ANTXR1 was not only aberrantly upregulated in GC tissues but also significantly correlated with depth of tumor invasion and disease stage, Lauren classification, histological grade, tumor size, and race, indicating that tumors with high expression of ANTXR1 are malignant and aggressive. Consistent with a previous study (Sotoudeh et al., 2019), our research also revealed that ANTXR1 is an independent prognostic indicator for GC patients and overexpression of ANTXR1 was significantly associated with poor clinical outcomes. Taken together, ANTXR1 is an outstanding candidate for prognostic biomarker and therapeutic target of GC patients.

Immunotherapy, emerging as one of the most promising methods for cancer therapy, has been applied to clinical treatment of several cancer types, and its efficacy is closely related to the TME. Increasing attention has recently been paid into the TME, which is composed of immune cells, stromal cells, and extracellular components (cytokines, chemokines, extracellular matrix, etc.) (Wu and Dai, 2017). Tumor cells are capable of interacting with immune cells and stromal cells in the TME by direct contact or through chemokine and cytokine signal transduction, which can result in the TME remodeling (Wu and Dai, 2017). The TME plays a crucial role not only in regulating the proliferation, apoptosis, and metastasis of malignant cells but also in impacting cancer (immuno-)therapeutic efficacy (Wu and Dai, 2017; Maman and Witz, 2018). It is thus of great value to identify promising biomarkers and therapeutic target that play an important role in TME. To some extent, immune score and stromal score represent the infiltration levels of immune and stromal cells within the TME in GC. Here, our results suggested that high ANTXR1 expression was strongly correlated with stromal and immune infiltration in GC. Furthermore, we found that ANTXR1 expression significantly correlates with stromal and immune cell infiltration in GC via TIMER and xCell. Interestingly, these infiltrating cells are mainly composed of stromal cells (such as CAFs) and myeloid cells (such as M2 macrophages). In line with that, with few exceptions, the majority of immunosuppressive factors are strongly positively correlated with ANTXR1 expression in GC patients. Additionally, we found that ANTXR1 can induce T cell exhaustion, which can lead to poor clinical outcomes and failure of immunotherapy of cancer. Recently, increasing evidence has confirmed that crosstalk between EMT-related determinants and the TME might prompt tumor invasion and immune evasion (Terry et al., 2017). Indeed, Cai et al. found that ANTXR1 was able to promote EMT in GC (Cai et al., 2020). Consistent with it, our results also revealed that patients with high ANTXR1 expression tumors harbor more mesenchymal characteristics and less epithelial features in GC. Therefore, comprehensively learning the relationship between ANTXR1 and TME might contribute to a deeper understanding of the intricate interaction between malignant cells and the immune system, thus facilitating the progression of individualized immunotherapeutic drugs and enhancing the efficiency of immunotherapy in GC patients.

Metastasis is the predominant cause of death in cancer patients. It is well-known that abnormal EMT activation could prompt tumor cell invasion, metastasis, and chemoresistance with cellular adhesion molecules especially E-cadherin concomitantly hindered, leading to dissemination of cancer cells to other organs of the body (Huang et al., 2015). The transforming growth factor-β (TGF-β) signaling pathway is a major driver of EMT (Hua et al., 2020). Of note, we also found that high expression of ANTXR1 in GC was significantly correlated with EMT and TGF-β signaling pathway via GSEA. These results indicate that ANTXR1 may promote immune evasion through EMT and TGF-β signaling pathway. Nevertheless, these analyses were conducted only based on genomic data and additional experiments are needed to further elucidate the specific mechanism of how ANTXR1 impact the TME in GC.

Chemotherapy resistance is also an important element that impacts outcomes of GC patients. Tumor cells that interact with immune or stromal cells can induce TME remodeling and function as a key player in promoting chemotherapy resistance (Russi et al., 2019). Our study revealed that ANTXR1 played a crucial role in TME remodeling. Thus, we conducted an analysis of whether the expression level of ANTXR1 can serve as an indicator of the sensitivity of GC patients to adjuvant chemotherapy. Results suggested that high ANTXR1 expression tumors are less sensitive to adjuvant chemotherapy. This further demonstrated that ANTXR1 might promote TME remodeling, especially affecting the elements of tumor stroma, which can lead to chemoresistance. TMB represents the total index of mutations in tumor samples and serves as a promising biomarker in immunotherapy (Chalmers et al., 2017). It was confirmed to be related to the sensitivity to immune checkpoint inhibitors in multiple tumors (Chalmers et al., 2017). In the current study, we found that GC patients with high ANTXR1 expression tumors have lower TMB, which indicated that patients with low ANTXR1 expression may be more sensitive to immune checkpoint inhibitor therapy. Overall, ANTXR1 could be an indicator not only of sensitivity to adjuvant chemotherapy but also of immunotherapy in GC patients.

## Conclusions

In summary, we comprehensively investigated the correlation between ANTXR1 and clinicopathological characteristics, and prognosis of GC. Importantly, our research investigated the immunological role that ANTXR1 plays in the TME of GC. Meanwhile, the relationship between ANTXR1 and TMB or chemotherapy was also studied. Our study described the crucial role that ANTXR1 plays in the TME of GC, highlighting how ANTXR1 interact with the immune microenvironment and may guide a more precise and individualized immunotherapeutic strategy for GC patients. However, further in-depth experimental validations are indispensable to elucidate the specific mechanism of how ANTXR1 affects the TME in GC. Based on the expression level of ANTXR1 in GC, clinicians could select currently available standard medicine or potential monoclonal antibodies that target ANTXR1 proteins to maximize the benefits to patients, if confirmed in prospective studies, which would improve the clinical outcomes of GC patients.

## Data Availability Statement

The data in this research are acquired from The Cancer Genome Atlas (TCGA) (https://www.cancer.gov/tcga) and Gene Expression Omnibus (GEO) (https://www.ncbi.nlm.nih.gov/geo/) database.

## Author Contributions

XH and YZ made contributions to the conception and design of the research. XH, YZ, and JZ integrated and analyzed the data. XH and JZ wrote the manuscript. YZ edited and altered the manuscript. All authors read and approved the final manuscript.

## Conflict of Interest

The authors declare that the research was conducted in the absence of any commercial or financial relationships that could be construed as a potential conflict of interest.

## Supplementary Material

Supplementary Figure 1 Heatmap of the representative differentially expressed genes in the two groups (high vs. low ANTXR1 expression).

Supplementary Figure 2 Enrichment plots from the gene set enrichment analysis (GSEA). **(A)** Enriched gene sets in C2 collection by samples of high ANTXR1 expression. Only several leading gene sets are shown in the plot. **(B)** Enriched gene sets in Hallmark collection by the high ANTXR1 expression samples. Only several leading gene sets were displayed in the plot.

## References

[B1] Ajani J. A. Lee J. Sano T. Janjigian Y. Y. Fan D. Song S. (2017). Gastric adenocarcinoma. Nat. Rev. Dis. Primers 3:17036 10.1038/nrdp.2017.36 28569272

[B2] Alcala S. Martinelli P. Hermann P. C. Heeschen C. Sainz B. J. (2019). The anthrax toxin receptor 1 (ANTXR1) is enriched in pancreatic cancer stem cells derived from primary tumor cultures. Stem Cells Int. 2019:1378639. 10.1155/2019/1378639 31191663 PMC6525821

[B3] Aran D. Hu Z. Butte A. J. (2017). xCell: digitally portraying the tissue cellular heterogeneity landscape. Genome Biol. 18:220. 10.1186/s13059-017-1349-1 29141660 PMC5688663

[B4] Bijlsma M. F. Sadanandam A. Tan P. Vermeulen L. (2017). Molecular subtypes in cancers of the gastrointestinal tract. Nat. Rev. Gastroenterol. Hepatol. 14, 333–342. 10.1038/nrgastro.2017.33 28400627

[B5] Blank C. U. Haining W. N. Held W. Hogan P. G. Kallies A. Lugli E.. (2019). Defining ‘T cell exhaustion’. Nat. Rev. Immunol. 19, 665–674. 10.1038/s41577-019-0221-9 31570879 PMC7286441

[B6] Bray F. Ferlay J. Soerjomataram I. Siegel R. L. Torre L. A. Jemal A. (2018). Global cancer statistics 2018: GLOBOCAN estimates of incidence and mortality worldwide for 36 cancers in 185 countries. CA Cancer J. Clin. 68, 394–424. 10.3322/caac.21492 30207593

[B7] Byrd T. T. Fousek K. Pignata A. Szot C. Samaha H. Seaman S. (2018). TEM8/ANTXR1-specific CAR T cells as a targeted therapy for triple-negative breast cancer. Cancer Res. 78, 489–500. 10.1158/0008-5472.CAN-16-1911 29183891 PMC5771806

[B8] Cai C. Dang W. Liu S. Huang L. Li Y. Li G.. (2020). Anthrax toxin receptor 1/tumor endothelial marker 8 promotes gastric cancer progression through activation of the PI3K/AKT/mTOR signaling pathway. Cancer Sci. 111, 1132–1145. 10.1111/cas.14326 31977138 PMC7156833

[B9] Chalmers Z. R. Connelly C. F. Fabrizio D. Gay L. Ali S. M. Ennis R.. (2017). Analysis of 100,000 human cancer genomes reveals the landscape of tumor mutational burden. Genome Med. 9:34. 10.1186/s13073-017-0424-2 28420421 PMC5395719

[B10] Chan T. A. Yarchoan M. Jaffee E. Swanton C. Quezada S. A. Stenzinger A.. (2019). Development of tumor mutation burden as an immunotherapy biomarker: utility for the oncology clinic. Ann. Oncol. 30, 44–56. 10.1093/annonc/mdy495 30395155 PMC6336005

[B11] Chaudhary A. Hilton M. B. Seaman S. Haines D. C. Stevenson S. Lemotte P. K.. (2012). TEM8/ANTXR1 blockade inhibits pathological angiogenesis and potentiates tumoricidal responses against multiple cancer types. Cancer Cell 21, 212–226. 10.1016/j.ccr.2012.01.004 22340594 PMC3289547

[B12] Cristescu R. Lee J. Nebozhyn M. Kim K. M. Ting J. C. Wong S. S.. (2015). Molecular analysis of gastric cancer identifies subtypes associated with distinct clinical outcomes. Nat. Med. 21, 449–456. 10.1038/nm.3850 25894828

[B13] Davies G. Rmali K. A. Watkins G. Mansel R. E. Mason M. D. Jiang W. G. (2006). Elevated levels of tumour endothelial marker-8 in human breast cancer and its clinical significance. Int. J. Oncol. 29, 1311–1317. 10.3892/ijo.29.5.1311 17016666

[B14] Gambardella V. Castillo J. Tarazona N. Gimeno-Valiente F. Martinez-Ciarpaglini C. Cabeza-Segura M.. (2020). The role of tumor-associated macrophages in gastric cancer development and their potential as a therapeutic target. Cancer Treat. Rev. 86:102015. 10.1016/j.ctrv.2020.102015 32248000

[B15] Gentles A. J. Newman A. M. Liu C. L. Bratman S. V. Feng W. Kim D.. (2015). The prognostic landscape of genes and infiltrating immune cells across human cancers. Nat. Med. 21, 938–945. 10.1038/nm.3909 26193342 PMC4852857

[B16] Hua W. Ten D. P. Kostidis S. Giera M. Hornsveld M. (2020). TGFbeta-induced metabolic reprogramming during epithelial-to-mesenchymal transition in cancer. Cell. Mol. Life Sci. 77, 2103–2123. 10.1007/s00018-019-03398-6 31822964 PMC7256023

[B17] Huang L. Wu R. L. Xu A. M. (2015). Epithelial-mesenchymal transition in gastric cancer. Am. J. Transl. Res. 7, 2141–2158. 26807164 PMC4697696

[B18] Huang L. Xu A. M. Liu S. Liu W. Li T. J. (2014). Cancer-associated fibroblasts in digestive tumors. World J. Gastroenterol. 20, 17804–17818. 10.3748/wjg.v20.i47.17804 25548479 PMC4273131

[B19] Kim S. T. Cristescu R. Bass A. J. Kim K. M. Odegaard J. I. Kim K.. (2018). Comprehensive molecular characterization of clinical responses to PD-1 inhibition in metastatic gastric cancer. Nat. Med. 24, 1449–1458. 10.1038/s41591-018-0101-z 30013197

[B20] Liu Y. Sethi N. S. Hinoue T. Schneider B. G. Cherniack A. D. Sanchez-Vega F.. (2018). Comparative molecular analysis of gastrointestinal adenocarcinomas. Cancer Cell 33, 721–735. 10.1016/j.ccell.2018.03.010 29622466 PMC5966039

[B21] Maman S. Witz I. P. (2018). A history of exploring cancer in context. Nat. Rev. Cancer 18, 359–376. 10.1038/s41568-018-0006-7 29700396

[B22] Maron S. B. Alpert L. Kwak H. A. Lomnicki S. Chase L. Xu D.. (2018). Targeted therapies for targeted populations: anti-EGFR treatment for EGFR-amplified gastroesophageal adenocarcinoma. Cancer Discov. 8, 696–713. 10.1158/2159-8290.CD-17-1260 29449271 PMC5984701

[B23] Mayakonda A. Lin D. Assenov Y. Plass C. Koeffler H. P. (2018). Maftools: efficient and comprehensive analysis of somatic variants in cancer. Genome Res. 28, 1747–1756. 10.1101/gr.239244.118 30341162 PMC6211645

[B24] Niccolai E. Taddei A. Prisco D. Amedei A. (2015). Gastric cancer and the epoch of immunotherapy approaches. World J. Gastroenterol. 21, 5778–5793. 10.3748/wjg.v21.i19.5778 26019442 PMC4438012

[B25] Ritchie M. E. Phipson B. Wu D. Hu Y. Law C. W. Shi W.. (2015). limma powers differential expression analyses for RNA-sequencing and microarray studies. Nucleic Acids Res. 43:gkv007. 10.1093/nar/gkv007 25605792 PMC4402510

[B26] Rmali K. A. Watkins G. Harrison G. Parr C. Puntis M. C. Jiang W. G. (2004). Tumour endothelial marker 8 (TEM-8) in human colon cancer and its association with tumour progression. Eur. J. Surg. Oncol. 30, 948–953. 10.1016/j.ejso.2004.07.023 15498639

[B27] Roma-Rodrigues C. Mendes R. Baptista P. V. Fernandes A. R. (2019). Targeting tumor microenvironment for cancer therapy. Int. J. Mol. Sci. 20:40840. 10.3390/ijms20040840 30781344 PMC6413095

[B28] Roviello G. Generali D. (2018). Pertuzumab therapy for HER2-positive metastatic gastric or gastro-oesophageal junction cancer. Lancet Oncol. 19, 1270–1272. 10.1016/S1470-2045(18)30512-6 30217673

[B29] Russi S. Verma H. K. Laurino S. Mazzone P. Storto G. Nardelli A.. (2019). Adapting and surviving: intra and extra-cellular remodeling in drug-resistant gastric cancer cells. Int. J. Mol. Sci. 20:3736. 10.3390/ijms20153736 31370155 PMC6695752

[B30] Samstein R. M. Lee C. H. Shoushtari A. N. Hellmann M. D. Shen R. Janjigian Y. Y.. (2019). Tumor mutational load predicts survival after immunotherapy across multiple cancer types. Nat. Genet. 51, 202–206. 10.1038/s41588-018-0312-8 30643254 PMC6365097

[B31] Sotoudeh M. Shakeri R. Dawsey S. M. Sharififard B. Ahmadbeigi N. Naderi M. (2019). ANTXR1 (TEM8) overexpression in gastric adenocarcinoma makes the protein a potential target of immunotherapy. Cancer Immunol. Immunother. 68, 1597–1603. 10.1007/s00262-019-02392-y 31520110 PMC7493837

[B32] Szot C. Saha S. Zhang X. M. Zhu Z. Hilton M. B. Morris K.. (2018). Tumor stroma-targeted antibody-drug conjugate triggers localized anticancer drug release. J. Clin. Invest. 128, 2927–2943. 10.1172/JCI120481 29863500 PMC6025988

[B33] Terry S. Savagner P. Ortiz-Cuaran S. Mahjoubi L. Saintigny P. Thiery J. P.. (2017). New insights into the role of EMT in tumor immune escape. Mol. Oncol. 11, 824–846. 10.1002/1878-0261.12093 28614624 PMC5496499

[B34] Torre L. A. Siegel R. L. Ward E. M. Jemal A. (2016). Global cancer incidence and mortality rates and trends–an update. Cancer Epidemiol. Biomarkers Prev. 25, 16–27. 10.1158/1055-9965.EPI-15-0578 26667886

[B35] Van Cutsem E. Sagaert X. Topal B. Haustermans K. Prenen H. (2016). Gastric cancer. Lancet 388, 2654–2664. 10.1016/S0140-6736(16)30354-3 27156933

[B36] Wu T. Dai Y. (2017). Tumor microenvironment and therapeutic response. Cancer Lett. 387, 61–68. 10.1016/j.canlet.2016.01.043 26845449

[B37] Yoshihara K. Shahmoradgoli M. Martinez E. Vegesna R. Kim H. Torres-Garcia W.. (2013). Inferring tumour purity and stromal and immune cell admixture from expression data. Nat. Commun. 4:2612. 10.1038/ncomms3612 24113773 PMC3826632

[B38] Yu G. Wang L. G. Han Y. He Q. Y. (2012). clusterProfiler: an R package for comparing biological themes among gene clusters. OMICS 16, 284–287. 10.1089/omi.2011.0118 22455463 PMC3339379

