# Supplementary figures and images for: ANTXR1 Is a Prognostic Biomarker and Correlates With Stromal and Immune Cell Infiltration in Gastric Cancer
Source: Front Mol Biosci. 2020 Dec 15;7:598221. doi: 10.3389/fmolb.2020.598221 (PMC7770144; doi:10.3389/fmolb.2020.598221)

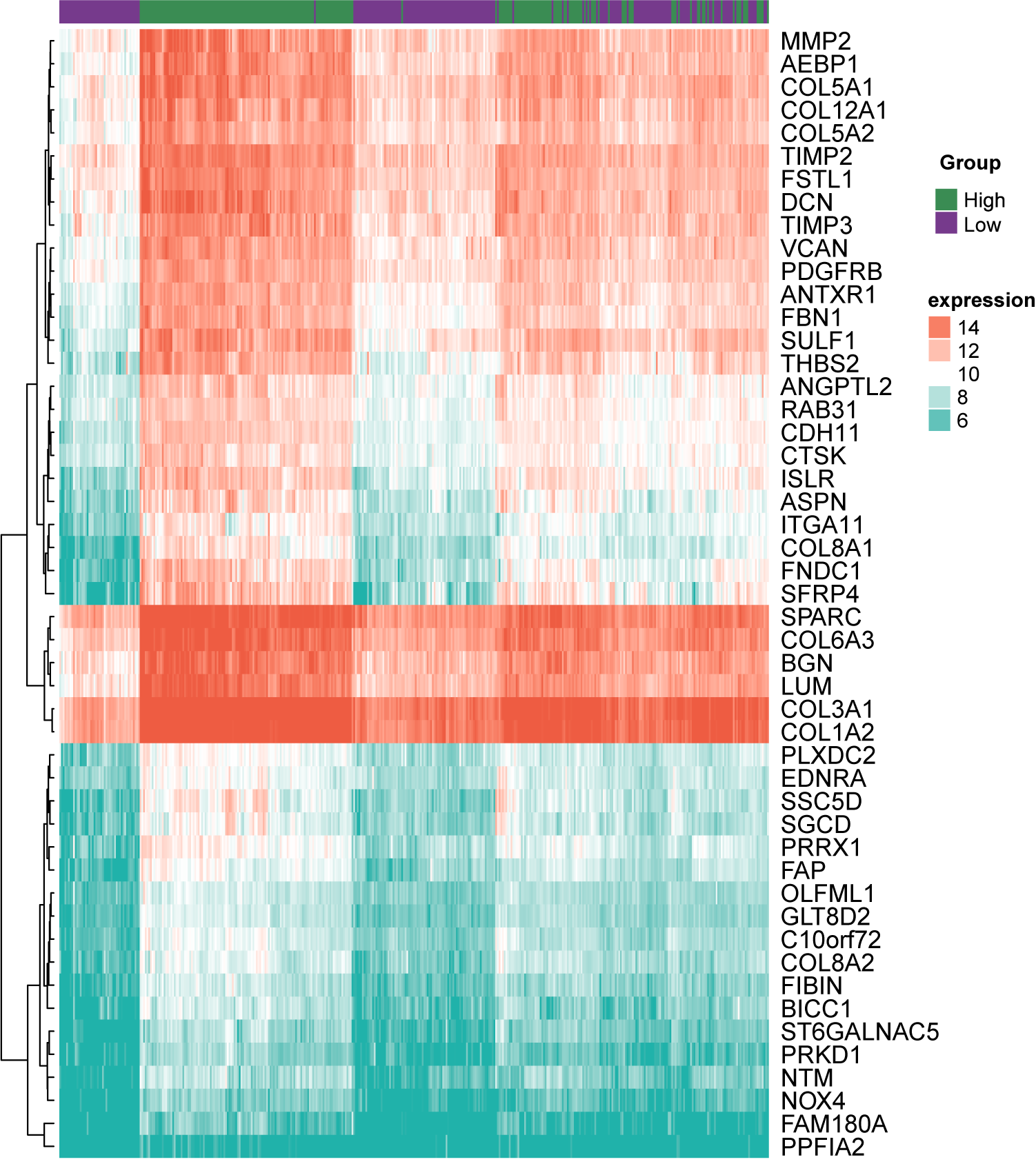

Supplement: Supplementary Figure 1 — Heatmap of the representative differentially expressed genes in the two groups (high vs. low ANTXR1 expression). [file Image_1.TIF]

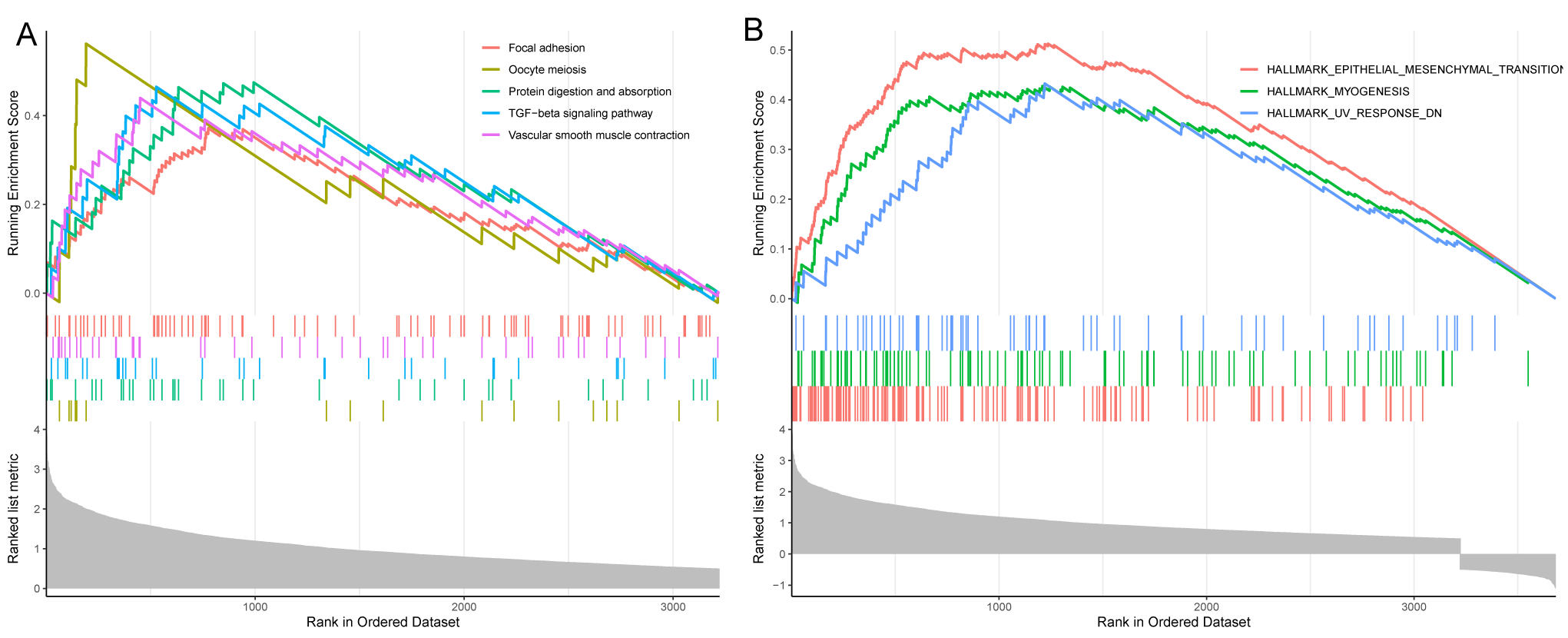

Supplement: Supplementary Figure 2 — Enrichment plots from the gene set enrichment analysis (GSEA). (A) Enriched gene sets in C2 collection by samples of high ANTXR1 expression. Only several leading gene sets are shown in the plot. (B) Enriched gene sets in Hallmark collection by the high ANTXR1 expression samples. Only several leading gene sets were displayed in the plot. [file Image_2.TIF]
